# Supplementary material for: Locus-level L1 DNA methylation profiling reveals the epigenetic and transcriptional interplay between L1s and their integration sites
Source: Cell Genom. 2024 Feb 2;4(2):100498. doi: 10.1016/j.xgen.2024.100498 (PMC10879037; doi:10.1016/j.xgen.2024.100498)
Supplement: Document S1. Figures S1–S7 [file mmc1.pdf]

Cell Genomics, Volume *4*

Supplemental information

Locus-level L1 DNA methylation profiling reveals the epigenetic and transcriptional interplay between L1s and their integration sites

Sophie Lanciano, Claude Philippe, Arpita Sarkar, David Pratella, Cécilia Domrane, Aurélien J. Doucet, Dominic van Essen, Simona Saccani, Laure Ferry, Pierre-Antoine Defossez, and Gael Cristofari

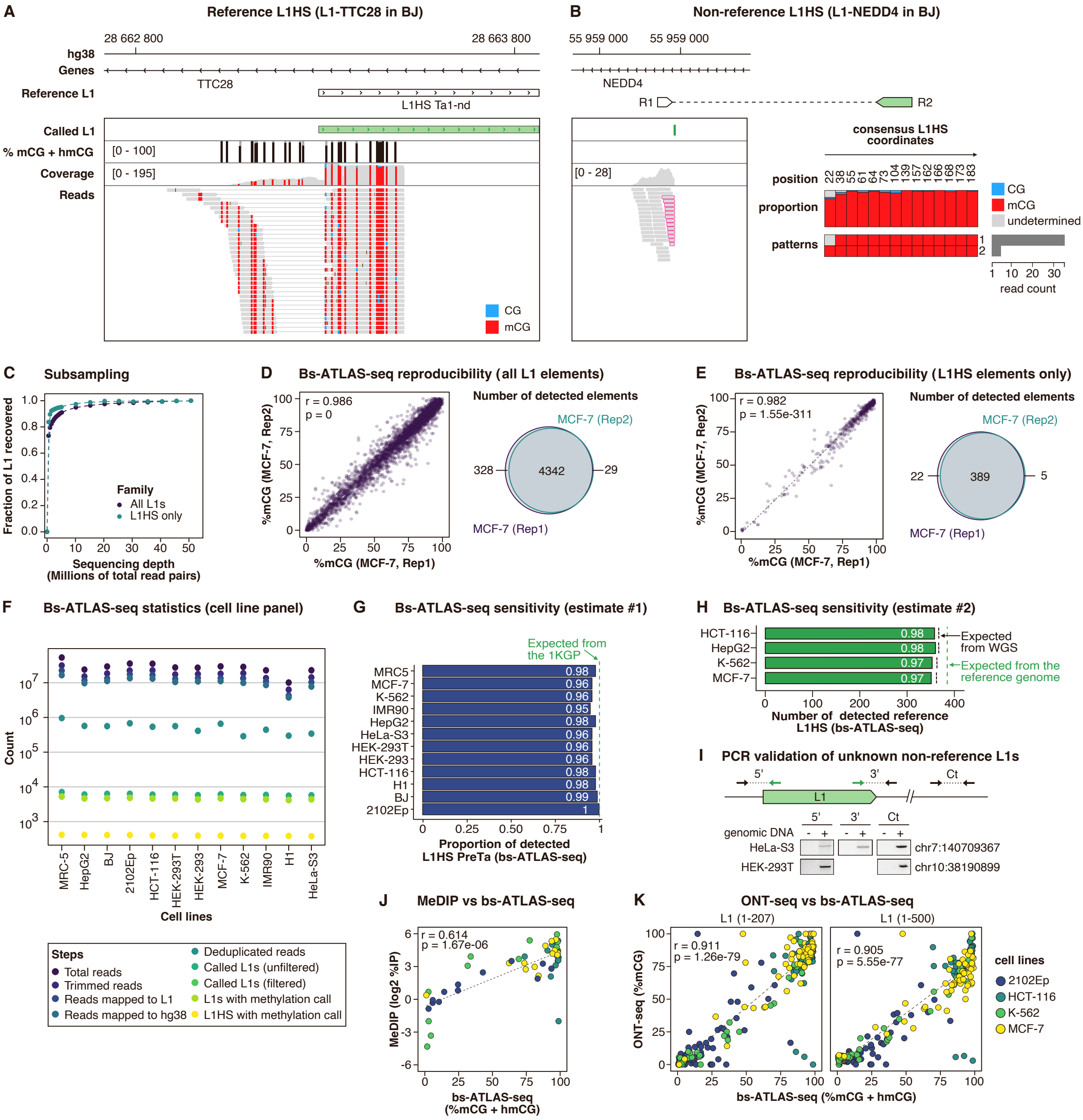

Figure S1, related to Figure 1 – Optimization, sequencing statistics and validation of bs-ATLAS-seq.

(**A**, **B**) Genome browser views of reference (A) and a non-reference (B) L1HS elements in the *TTC28* and *NEDD4* genes, respectively (BJ foreskin fibroblasts). CpG methylation is indicated by vertical bars (grey for the site, black for methylation percentage). Coverage and reads tracks show non-methylated (blue) and methylated (mCG, red) CpG sites. For non-reference L1HS (B), only the genomic region covered by read #1 (R1) is visible. Pink frames highlight soft-clipped reads supporting the 5' L1 junction. The proportion of mCG at each site and the frequency of the most common methylation patterns deduced from read #2 (R2) are indicated on the charts (right). CpG positions are relative to L1HS consensus sequence.

(**C**) Subsampling. Computational down-sampling of high depth bs-ATLAS-seq sequencing data (MCF7) shows that L1 recovery reaches a plateau above 10 million of total read pairs. Thus, all samples were subsequently sequenced to a depth greater than 10 million of total reads.

(**D, E**) Reproducibility of bs-ATLAS-seq. Two independent libraries of MCF-7 (from two subsequent MCF-7 passages) and sequencing runs are compared with respect to L1 elements of all families (D) or to L1HS elements only (E). Replicate 1 (MCF7, Rep1) was down sampled to the sequencing depth of replicate 2 (MCF7, Rep2) for comparison purpose. Left panels: Correlation of methylation levels for shared detected L1 loci with r and p representing Pearson correlation coefficient and p-value, respectively (D: n=4,342; and E: n=389). Right panels: Venn diagram showing the overlap of detected L1 loci between the two libraries.

(**F**) Statistics of bs-ATLAS-seq for the 12-cell line panel. See also **Table S1**.

(**G**) Evaluation of bs-ATLAS-seq sensitivity using reference L1HS-PreTa elements defined as fixed in the human population by the 1000 Genomes Project (1KGP). Sensitivity: 97.2 ± 1.7% (mean ± SD).

(**H**) Validation of true negative L1HS elements and determination of bs-ATLAS-seq sensitivity using publicly available whole genome sequencing (WGS) data from four cell lines. Sensitivity: 97.6 ± 2.1% (mean ± SD).

(**I**) PCR validation of unknown non-reference insertions. PCR was done with genomic DNA of the indicated cell lines (+) or with water as non-template control (-). The insertion in chr10 is pericentromeric and embedded in other repeats. Therefore, only primers to amplify the 5' junction could be designed. Ct, unrelated locus used as PCR control.

(**J**) DNA methylation level of selected L1 elements was profiled using an antibody-based enrichment of DNA methylation (MeDIP) and compared with bs-ATLAS-seq data (Pearson correlation, n=52; r and p, correlation coefficient and p-value, respectively). The DNA methylation level of MeDIP data is expressed as log2 of the percentage of immunoprecipitation (log2 %IP). See also **Table S3**.

(**K**) Comparison of bs-ATLAS-seq with PCR-free targeted sequencing and methylation calling by Oxford Nanopore Technology sequencing (ONT-seq) for the L1 region common to both methods (1-207) or the full CpG island (1-500) (Pearson correlation, n=205; r and p, correlation coefficient and p-value, respectively). See also **Table S6**.

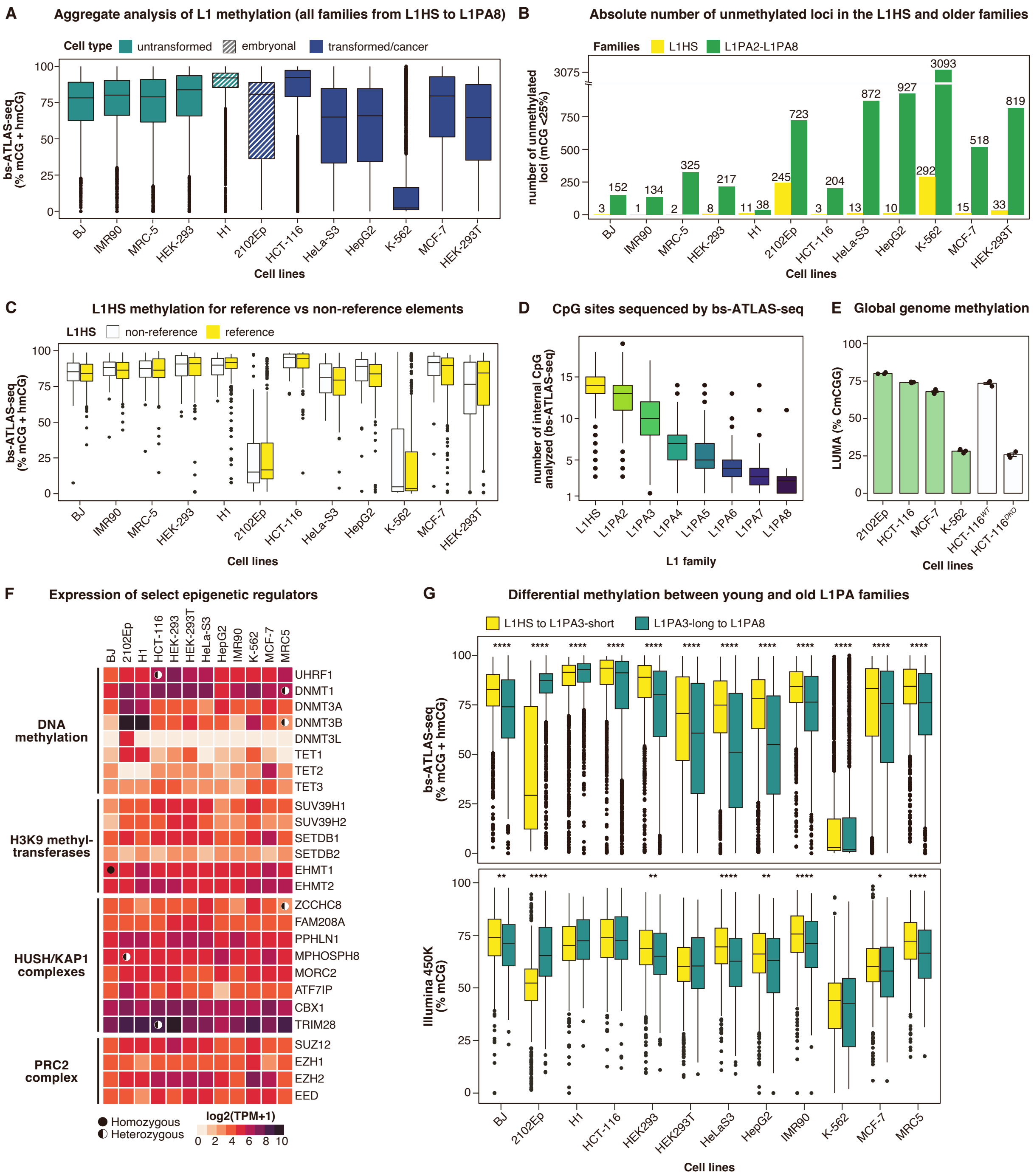

Figure S2, related to Figure 2 – L1 and global DNA methylation, and epigenetic effector expression in a panel of human cell lines.

(**A**) Aggregate DNA methylation level of the L1 promoter for all L1 elements detected by bs-ATLAS-seq (L1HS to L1PA8) across normal (green), embryonal (hatched), or transformed and cancer (blue) cells.

(**B**) Barplot indicating the absolute numbers of unmethylated L1 copies (% mCG<25% according to bs-ATLAS-seq) across the different cell lines, for L1HS (light green) and older copies (L1PA2 to L1PA8, dark green).

(**C**) Comparison of methylation levels for reference and non-reference L1HS elements across the different cell lines. Differences are not significant (two-sided Wilcoxon rank-sum test).

(**D**) Number of CpGs per element analyzed by bs-ATLAS-seq for each L1 family.

(**E**) Genome-wide global CpG methylation measured by LUminometric Methylation Assay (LUMA). A subset of the cell line panel assayed by bs-ATLAS-seq showing low- or high- levels of L1HS methylation were tested for genome-wide global CpG methylation by LUMA (green). HCT-116 DKO and WT refers to a double *DNMT1* and *DNMT3B* knock-out, and its parental cell line, respectively (white). These additional cell lines were used as controls in the LUMA assay. Bars represent the average percentage of methylated CCGG sites (mean ± sem, n=3 technical replicates).

(**F**) Heatmap of epigenetic effector expression across the different cell lines. TPM, transcripts per million of reads. Superimposed circles indicate homozygous (full circle) or heterozygous (half circle) variants detected by the VEP tool in RNA-seq data. Note that rare (AF < 0.01 in the global 1KGP population) and predicted as probably damaging (using the PolyPhen-2 tool) variants are represented, as well as variants with unknown AF and PolyPhen prediction. See **Table S4** for the full list of called variants.

(**G**) Comparison of methylation levels for young (L1HS to L1PA3-short) and older (L1PA3-long to L1PA8) L1PA elements across the different cell lines using bs-ATLAS-seq or BeadChip arrays. Top: bs-ATLAS-seq (each data point is an individual L1 element), bottom: 450K Illumina BeadChip arrays (each data point is an individual L1 probe). Publicly available 450K Illumina BeadChip array datasets are listed in **Table S5**.

In panels (A), (C), (D) and (G), boxplots represent the median and interquartile range (IQR) ± 1.5 * IQR (whiskers). Outliers beyond the end of the whiskers are plotted individually. *$p<0.05$, **$p<0.01$, ***$p<0.001$, and ****$p<0.0001$, two-sided Wilcoxon rank-sum test.

See also **Table S2**, **Table S4** and **Table S5**.

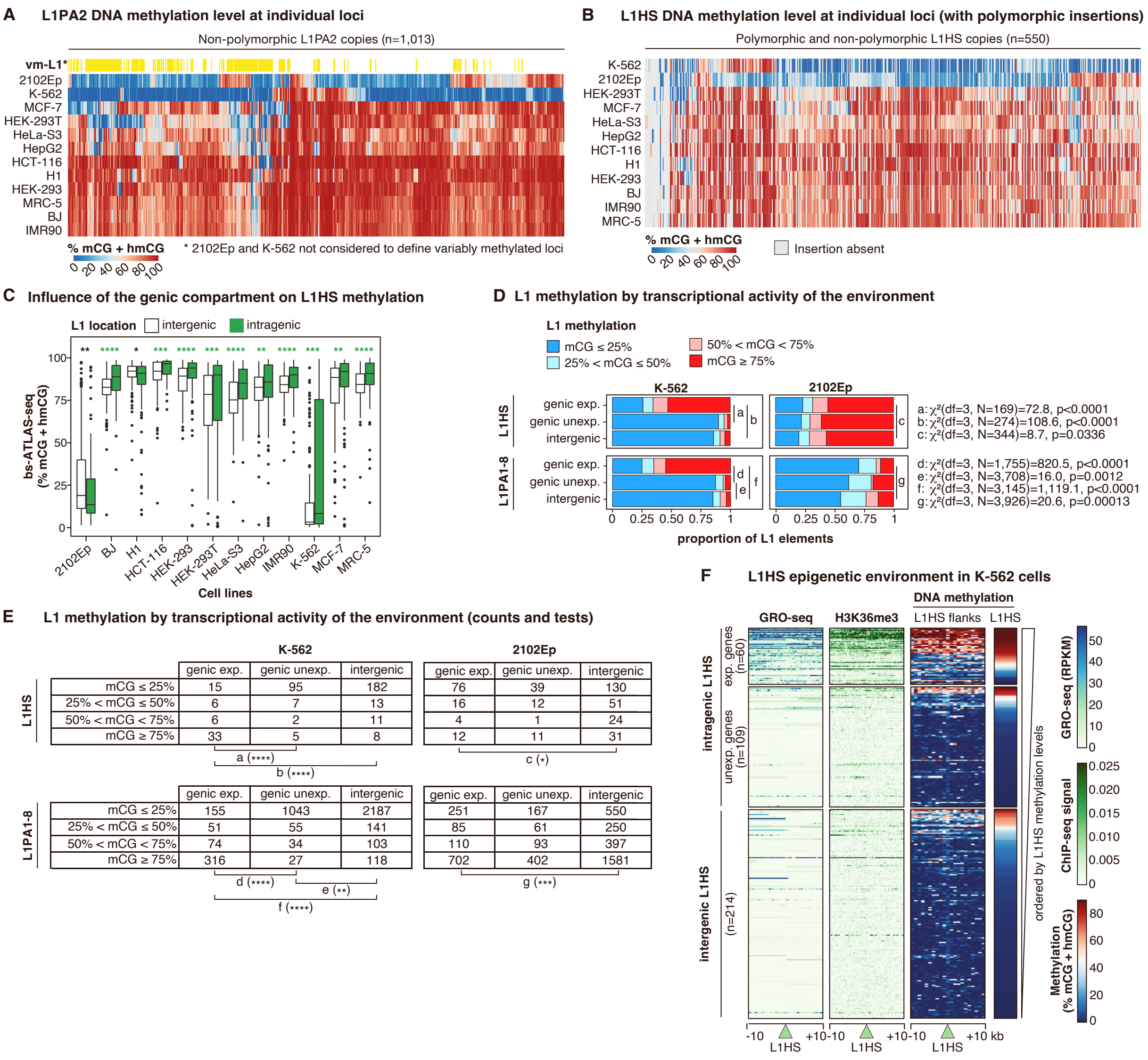

L1HS

	K-562 genic exp.	K-562 genic unexp.	K-562 intergenic	2102Ep genic exp.	2102Ep genic unexp.	2102Ep intergenic
mCG ≤ 25%	15	95	182	76	39	130
25% < mCG ≤ 50%	6	7	13	16	12	51
50% < mCG < 75%	6	2	11	4	1	24
mCG ≥ 75%	33	5	8	12	11	31

a (****) b (****) c (*)

L1PA1-8

	K-562 genic exp.	K-562 genic unexp.	K-562 intergenic	2102Ep genic exp.	2102Ep genic unexp.	2102Ep intergenic
mCG ≤ 25%	155	1043	2187	251	167	550
25% < mCG ≤ 50%	51	55	141	85	61	250
50% < mCG < 75%	74	34	103	110	93	397
mCG ≥ 75%	316	27	118	702	402	1581

d (****) e (**) f (****) g (***)

Figure S3, related to Figure 3 – Methylation levels of individual L1s and their link with transcription.

(**A**) Heatmap of bs-ATLAS-seq methylation levels (% mCG + hmCG) for individual L1PA2 loci across cell lines. Vm-L1, variably-methylated L1 loci.

(**B**) Heatmap of bs-ATLAS-seq methylation levels (% mCG + hmCG) displaying values for both reference and non-reference L1HS across cell lines. When an insertion is absent in a given cell line, the heatmap cell is colored in grey.

(**C**) Comparison of methylation levels for intra- (green) vs inter-genic (white) L1HS elements. Boxplots represent the median and interquartile range (IQR) ± 1.5 * IQR (whiskers). Outliers beyond the whiskers are plotted individually. *p < 0.05, **p<0.01, ***p < 0.001, and ****p < 0.0001 (black for decrease; green for increase), two-sided Wilcoxon rank-sum test, each L1 being considered as an observation.

(**D**) Distribution of L1 elements into methylation level categories based on genic environment in K-562 (left) or 2102Ep (right) cells. Note that L1PA1 is synonymous with L1HS.

(**E**) Counts and tests related to **Figure S3D**. Differences between 2 genomic environments were evaluated by chi-squared tests (letters), with their statistics shown in **Figure S3D**. *p < 0.05, **p < 0.01, ***p < 0.001, ****p < 0.0001.

(**F**) Heatmaps illustrating nascent transcription (GRO-seq), H3K36me3 histone modifications (ChIP-seq), and DNA methylation (whole genome bisulfite sequencing, WGBS), in 10 kb-windows upstream and downstream L1HS elements (green triangle). Loci are separated according to their position relative to genes (left), separating expressed and unexpressed genes, and sorted by decreasing L1 methylation levels (bs-ATLAS-seq, right). A similar heatmap including all families (from L1HS to L1PA8) is shown in **Figure 3C**.

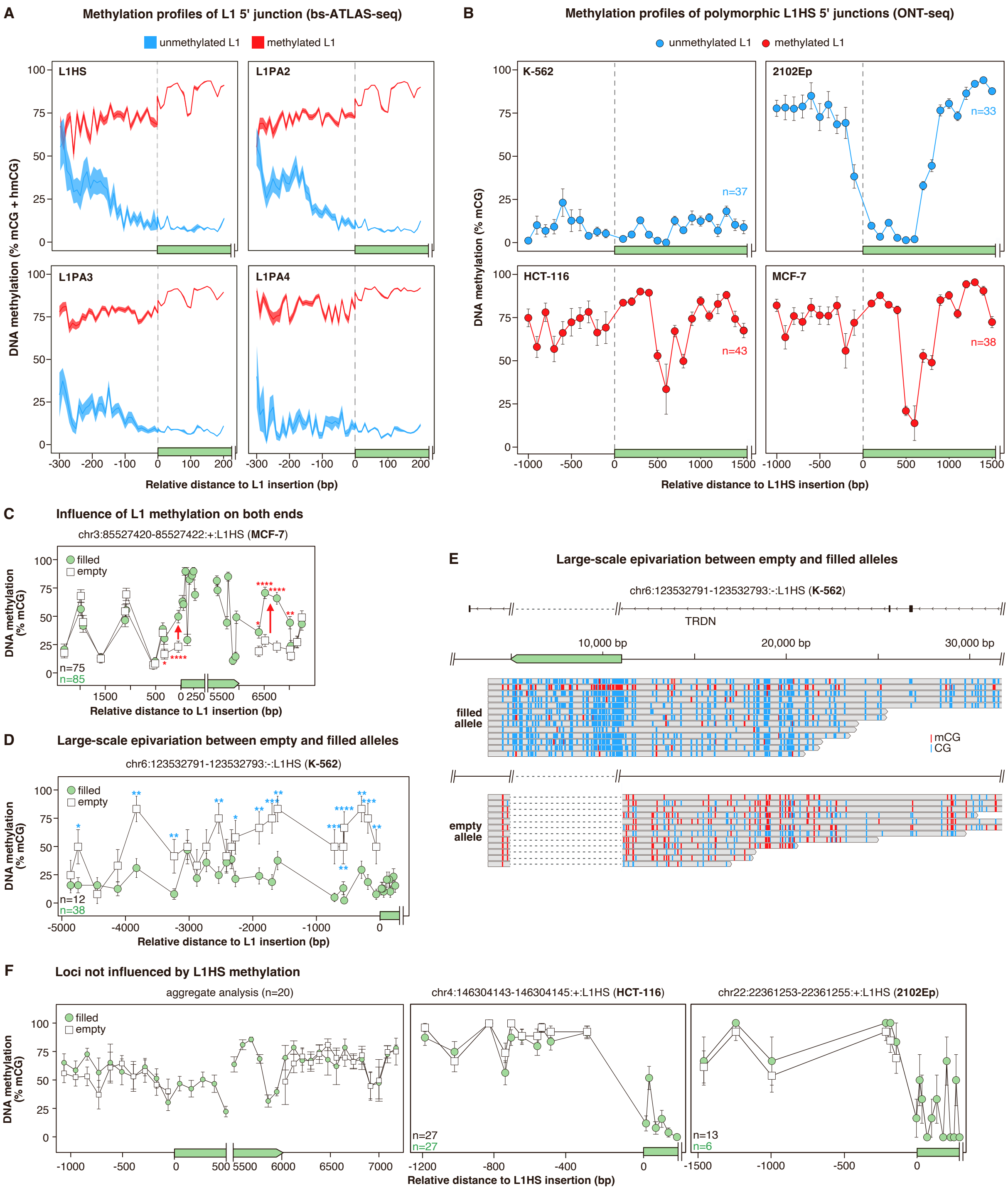

Figure S4, related to Figure 4 – DNA methylation profiles of L1s and their target loci.

(**A**) DNA methylation profiles of methylated (mCG ≥ 75%, red) and unmethylated (mCG ≤ 25%, blue) L1 (L1HS to L1PA4) 5' junctions obtained by bs-ATLAS-seq data. Data represent average DNA methylation levels in 10 bp-bins aggregated from the 12 cell lines (mean ± 95% C.I.).

(**B**) DNA methylation profiles of methylated (mCG ≥ 75%; red) and unmethylated (mCG ≤ 25%; blue) L1HS 5' junctions obtained by ONT-seq in 4 different cell lines (K-562, 2102Ep, HCT-116, MCF-7). Note that for the sake of comparison, the distinction of methylated vs unmethylated L1 is based on the first 15 CpG, as for bs-ATLAS-seq. Given the small numbers, unmethylated L1 in HCT-116 and MCF-7 (n=3 and n=3, respectively), and methylated L1s in K-562 (n=4) were not plotted. Data points represent average mCG levels in 100 bp-bins for each cell line (mean ± s.d.). n represents the number of loci.

(**C**) Example of locus with DNA methylation spreading from L1 to the external flanks. Red arrows denote hypermethylation relative to the empty locus. Difference of methylation between the empty and filled alleles was

tested by a two-sided Wilcoxon rank-sum test, with each read being an observation. *$p < 0.05$, **$p < 0.01$, ***$p < 0.001$, and ****$p < 0.0001$, n represents the number of reads per allele.

(**D, E**) Large-scale allele-specific epivariation associated with an L1 insertion. (D) Methylation levels and (E) genome browser view (Top: Filled allele; Bottom: Empty allele). The L1 insertion is depicted as a green solid arrow. Methylated and unmethylated CpG are indicated in red and blue, respectively. Difference of methylation between the empty and filled alleles was tested by a two-sided Wilcoxon rank-sum test, with each read being an observation. *$p < 0.05$, **$p < 0.01$, ***$p < 0.001$, and ****$p < 0.0001$, n represents the number of reads per allele.

(**F**) Loci not influenced by L1 methylation state. Left: Average DNA methylation levels in 100 bp-bins (n=20, mean ± s.d.). Middle and right: Examples of loci not influenced by L1 methylation. The difference in methylation between the empty and filled alleles was tested using a two-sided Wilcoxon rank-sum test. Each locus (left panel) or each read (middle and right panels) was considered an observation; n represents the number of reads per allele (empty: in black; filled: in green). The test showed no significant difference between the two alleles at any position.

For (A) to (D), and (F), the x-axis represents the relative distance to L1 5' end (green) and the y-axis the percentage of DNA methylation. For (C), (D) and (F), the empty (white squares) and filled (green circles) alleles are overlaid.

See also **Table S2** and **Table S6**.

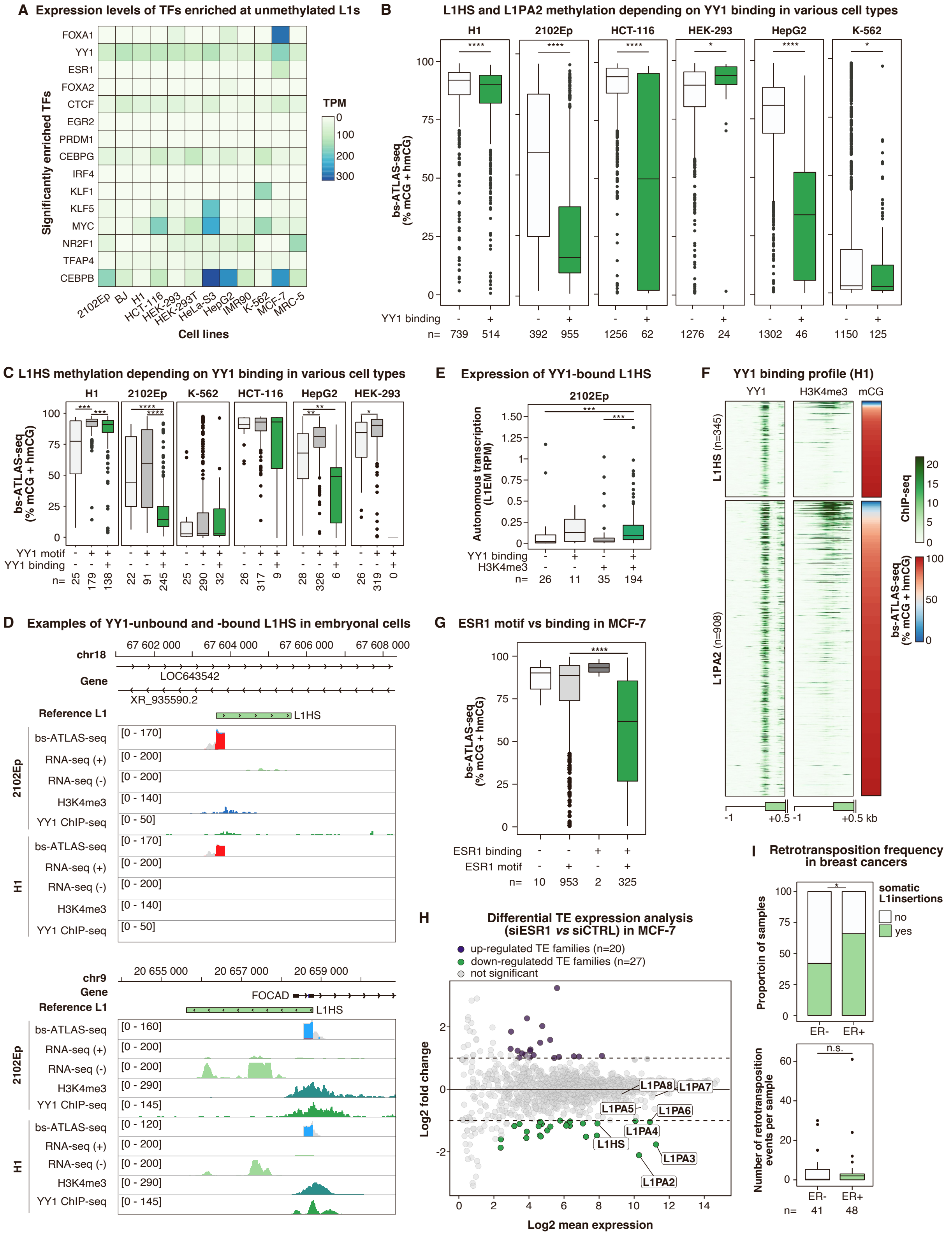

Figure S5, related to Figure 5 – Association of unmethylated L1HS and L1PA2 with transcription factors.

(**A**) Heatmap of TF expression levels across cell lines, restricted to TF identified in **Figure 5B**. FOXA1 and ESR1 are more expressed in MCF-7 as compared to other cell types whereas YY1 is more ubiquitously expressed, even if it predominantly binds to L1 elements in embryonal cells (H1 and 2102Ep) (see **Figure 5E** and panel B). TPM, transcripts per million.

(**B**) Comparison of DNA methylation levels of L1HS and L1PA2 elements bound (green) or unbound (white) by YY1 in embryonic cell lines (H1 and 2102Ep) and other cell lines (K-562, HCT116, HepG2, HEK-293). N: Number of L1HS copies in each subset.

(**C**) Comparison of DNA methylation levels of L1HS elements with (+) or without (-) YY1 binding motifs in their 5' UTR, and actually bound (+) or not (-) by YY1 in embryonal cell lines (H1 and 2102Ep) and other cell lines for which matched YY1 ChIP-seq were also publicly available (K-562, HCT116, HepG2, HEK-293). N: Number of L1HS copies in each subset. The data are identical to those presented in Figure 5E, but the loci unbound by YY1 were divided into two categories based on the presence or absence of the YY1 motif.

(**D**) Genome browser view of two example L1HS loci with distinct promoter DNA methylation profiles (bs-ATLAS-seq), showing poly(A)+ RNA-seq, YY1 and H3K4me3 ChIP-seq data in H1 and 2102Ep cells. Top: Locus in chromosome 18, the YY1 signal is close to the background level, the L1HS element is hypermethylated and non-expressed. Both cell lines have similar profiles. Bottom: Locus in chromosome 9, a strong YY1 peak is detected in H1 and 2102Ep cells, where the L1HS element is completely unmethylated and robustly expressed.

(**E**) Expression levels of L1HS element bound (+) or unbound (-) by YY1 or H3K4me3 (2102Ep cells). Locus-level expression was estimated by L1EM. N: Number of L1HS copies in each subset.

(**F**) Heatmap of L1 methylation (bs-ATLAS-seq), and YY1 and H3K4me3 binding (ChIP-seq), at L1HS and L1PA2 5' junction (H1 cells). Loci are sorted by increasing levels of L1 methylation. ChIP-seq signal represents the number of normalized reads per 10-bp bin.

(**G**) Comparison of DNA methylation levels of L1HS and L1PA2 loci with (+) or without (-) ESR1 binding motif in their 5' UTR and actually bound (+) or not (-) by ESR1 in MCF-7 cells. Two L1s without internal ESR1 binding motif bound by ESR1 (dark grey) show an upstream motif (<300 bp from the L1).

(**H**) Differential expression of transposable element (TE) families upon *ESR1* knockdown (MCF-7 cells) quantified by TEtranscripts [S1] (data from GSE153250). Each data point represents an aggregated TE family. TE families found significantly up- or down-regulated upon ESR1 knockdown are colored in purple and green, respectively, and data points corresponding to the L1HS to L1PA8 families are labelled (of which L1HS to L1PA6 are downregulated).

(**I**) Somatic L1 retrotransposition in breast cancer according to the estrogen receptor (ER) status in PCAWG samples. Top: Proportion of cancer samples with at least one somatic L1 insertion. *$p < 0.05$, chi-squared test: χ^2(df=1, N=344)=5.1908, p=.02271). Bottom: Number of somatic L1 retrotransposition events per sample. ER status was obtained from [S2] and somatic L1 retrotransposition events were identified in [S3]. N.s. non-significant, two-sided Wilcoxon rank-sum test; n: Number of samples in each group.

In panels (B), (C), (E), (G) and (I, bottom), boxplots represent the median and interquartile range (IQR) ± 1.5 * IQR (whiskers). Outliers beyond the whiskers are plotted individually. *$p < 0.05$, **$p < 0.01$, ***$p < 0.001$, and ****$p < 0.0001$, two-sided Wilcoxon rank-sum test, with each L1 locus being considered as an observation.

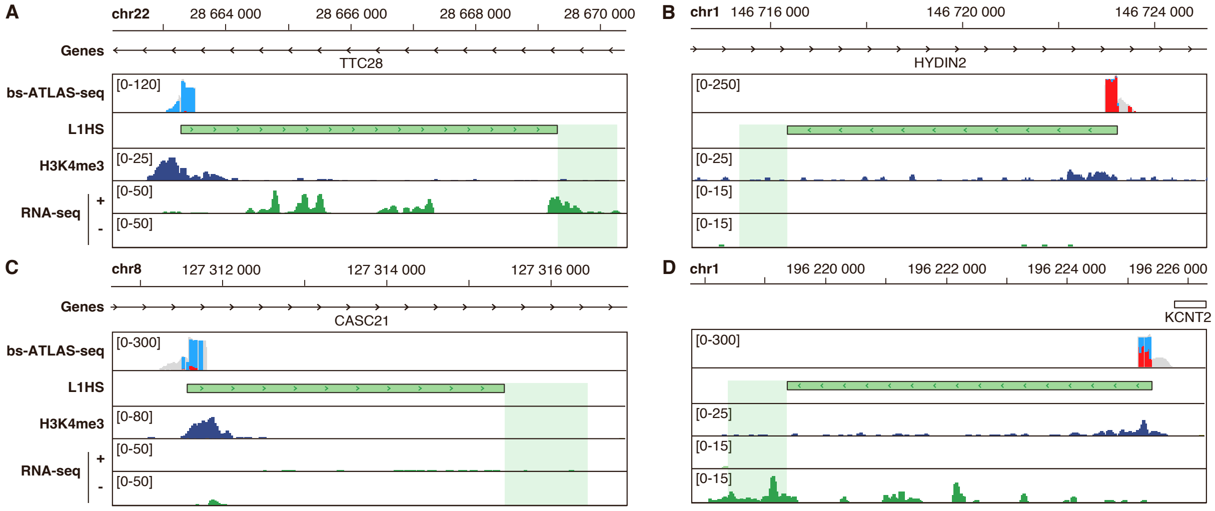

Figure S6, related to Figure 6 – Genome browser views of L1HS loci with different DNA methylation and expression profiles in MCF-7 cells.

(**A**) An unmethylated and expressed L1HS.

(**B**) A methylated and unexpressed L1HS.

(**C**) An unmethylated and unexpressed L1HS (note that the L1 antisense promoter appears to be active).

(**D**) A half-methylated and expressed L1HS.

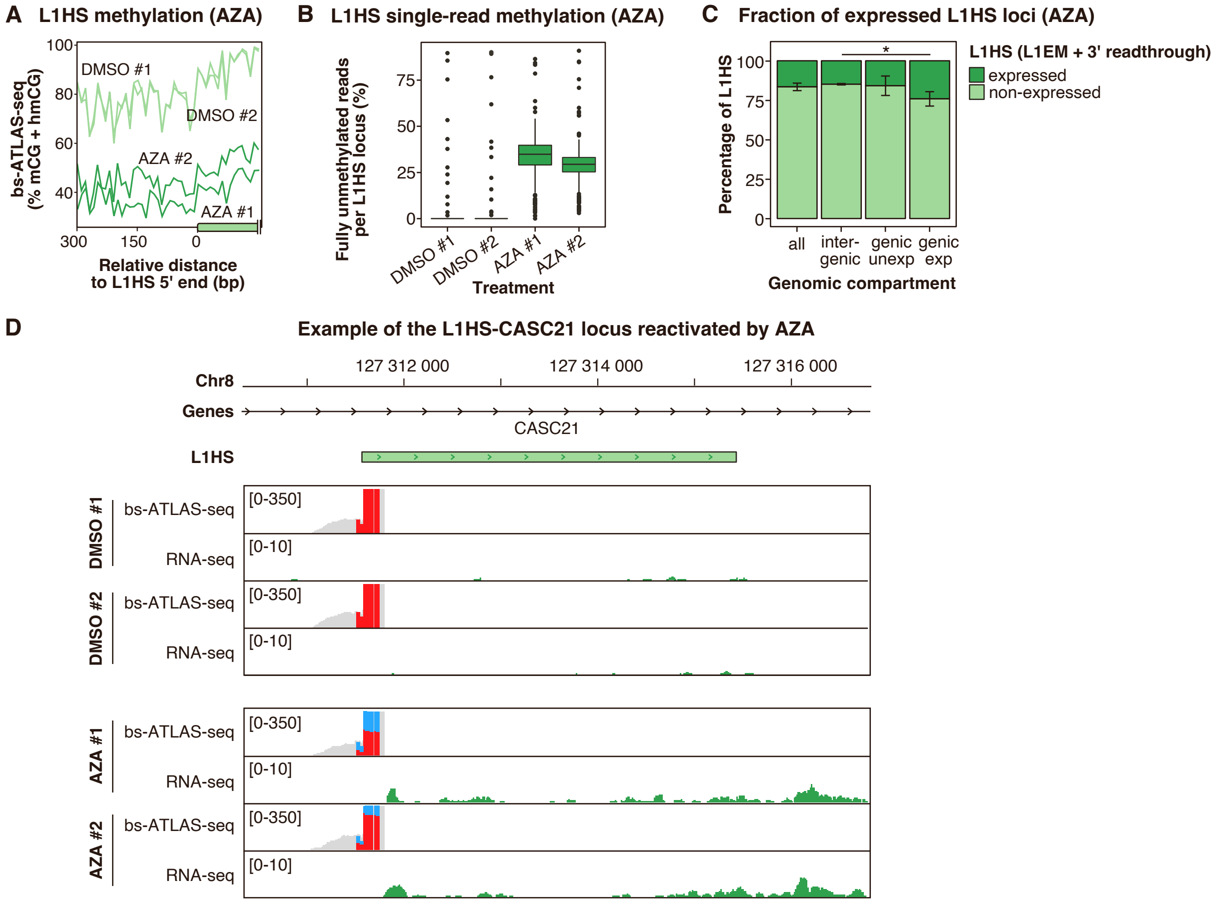

Figure S7, related to Figure 7 – Effect of acute DNA demethylation by 5-aza-2-deoxycytidine treatment on L1HS expression.

(**A**) Metaplot of L1 DNA methylation profiles (bs-ATLAS-seq) at the L1HS promoter and upstream flanking region (300 bp) in HCT-116 treated with 5-aza-2-deoxycytidine (two replicates: AZA #1 and AZA #2; dark green) or DMSO (two replicates: DMSO #1 and DMSO #2; light green).

(**B**) Fraction of fully unmethylated reads in 5-aza- or DMSO-treated HCT-116 cells. Boxplots represent the median and interquartile range (IQR) ± 1.5 * IQR (whiskers). Outliers beyond the whiskers are plotted individually. See (C) for legend.

(**C**) Proportion of unexpressed (light green) and expressed (dark green) L1HS loci upon 5'-aza treatment by genic environment in HCT-116 cells. *$p < 0.05$, chi-squared test: χ^2(df=1, N=280)=5.2, p=0.023.

(**D**) Genome browser view of the *CASC21* locus in cells treated (AZA) or not (DMSO) by 5-aza with L1 methylation (bs-ATLAS-seq) and expression (poly(A)+ RNA-seq).

Supplemental references

S1. Jin, Y., Tam, O.H., Paniagua, E., and Hammell, M. (2015). TEtranscripts: a package for including transposable elements in differential expression analysis of RNA-seq datasets. Bioinformatics *31*, 3593–3599. 10.1093/bioinformatics/btv422.

S2. Thennavan, A., Beca, F., Xia, Y., Garcia-Recio, S., Allison, K., Collins, L.C., Tse, G.M., Chen, Y.-Y., Schnitt, S.J., Hoadley, K.A., et al. (2021). Molecular analysis of TCGA breast cancer histologic types. Cell Genomics *1*, 100067. 10.1016/j.xgen.2021.100067.

S3. Rodríguez-Martín, B., Alvarez, E.G., Baez-Ortega, A., Zamora, J., Supek, F., Demeulemeester, J., Santamarina, M., Ju, Y.S., Temes, J., Garcia-Souto, D., et al. (2020). Pan-cancer analysis of whole genomes identifies driver rearrangements promoted by LINE-1 retrotransposition. Nat. Genet. *52*, 306–319. 10.1038/s41588-019-0562-0.
