# Supplementary material for: Locus-level L1 DNA methylation profiling reveals the epigenetic and transcriptional interplay between L1s and their integration sites
Source: Cell Genom. 2024 Feb 2;4(2):100498. doi: 10.1016/j.xgen.2024.100498 (PMC10879037; doi:10.1016/j.xgen.2024.100498)
Supplement: Data S1. Single-molecule methylation patterns of full-length and 3′-truncated L1 obtained by bs-ATLAS-seq, related to Figure 1 — Can be downloaded from https://doi.org/10.5281/zenodo.7097318. Coordinates are related to the hg38 reference genome (REF insertions) or to L1HS consensus sequence (NONREF insertions). [file mmc2.zip › BJ.bs-atlas-seq.L1HS.html]

Methylation patterns


# Methylation of L1HS elements found in BJ (both full-length and 3'-truncated)

- Graphs
- Settings
- Order
- Info

### Visualisation settings

#### Methylation patterns

Methylation site spacing

Show methylation site spacing

Methylation site spacing factor

Sort methylation patterns by

epiallele frequency
degree of methylation

Sort direction (left to right)

descending
ascending

Pattern read threshold (percent)

PNG file save scale factor

Cell size (pixels)

Scale pattern intensity

false
true

#### Histogram

Histogram scaling

linear
log

Histogram visible

true
false

Histogram height (pixels)

Histogram units

absolute
percent

#### Colour

Methylated site

Unmethylated site

Unknown site

Histogram

### Amplicon order

Drag the amplicon names up or down to reorder their display.

- ↕ chr1:34566055-34572105:-:L1HS:REF
- ↕ chr1:67078891-67084915:-:L1HS:REF
- ↕ chr1:68736693-68740136:-:L1HS:REF
- ↕ chr1:77000000-77005515:-:L1HS:REF
- ↕ chr1:80939203-80945257:-:L1HS:REF
- ↕ chr1:84052389-84058406:-:L1HS:REF
- ↕ chr1:85748519-85754548:+:L1HS:REF
- ↕ chr1:85927067-85933100:+:L1HS:REF
- ↕ chr1:86679080-86685111:-:L1HS:REF
- ↕ chr1:102103367-102103369:+:L1HS:NONREF
- ↕ chr1:104770247-104776278:-:L1HS:REF
- ↕ chr1:104843833-104849864:-:L1HS:REF
- ↕ chr1:113497220-113500000:+:L1HS:REF
- ↕ chr1:118852351-118858380:+:L1HS:REF
- ↕ chr1:121532230-121538261:-:L1HS:REF
- ↕ chr1:146717192-146723218:-:L1HS:REF
- ↕ chr1:166243053-166249084:-:L1HS:REF
- ↕ chr1:174590323-174596379:-:L1HS:REF
- ↕ chr1:180866811-180872843:-:L1HS:REF
- ↕ chr1:184845616-184851641:+:L1HS:REF
- ↕ chr1:187343764-187349794:-:L1HS:REF
- ↕ chr1:187597671-187603699:+:L1HS:REF
- ↕ chr1:193717837-193723892:-:L1HS:REF
- ↕ chr1:196219370-196225402:-:L1HS:REF
- ↕ chr1:197707714-197713746:+:L1HS:REF
- ↕ chr1:197802331-197803141:+:L1HS:REF
- ↕ chr1:209913771-209919823:-:L1HS:REF
- ↕ chr1:216485645-216487614:+:L1HS:REF
- ↕ chr1:218009227-218015252:-:L1HS:REF
- ↕ chr1:220245155-220246008:+:L1HS:REF
- ↕ chr1:237019467-237025494:-:L1HS:REF
- ↕ chr1:237075264-237081293:+:L1HS:REF
- ↕ chr1:239623498-239629523:-:L1HS:REF
- ↕ chr2:11000000-11002136:-:L1HS:REF
- ↕ chr2:16593725-16599758:-:L1HS:REF
- ↕ chr2:22968126-22968128:-:L1HS:NONREF
- ↕ chr2:36112506-36118537:-:L1HS:REF
- ↕ chr2:36343098-36343100:-:L1HS:NONREF
- ↕ chr2:41824275-41824277:+:L1HS:NONREF
- ↕ chr2:71411474-71417501:-:L1HS:REF
- ↕ chr2:81870918-81870920:+:L1HS:NONREF
- ↕ chr2:86655238-86661268:-:L1HS:REF
- ↕ chr2:102566355-102572385:-:L1HS:REF
- ↕ chr2:112503812-112509845:-:L1HS:REF
- ↕ chr2:126178048-126184064:-:L1HS:REF
- ↕ chr2:128816413-128816415:+:L1HS:NONREF
- ↕ chr2:129415687-129417400:-:L1HS:REF
- ↕ chr2:129685897-129687022:-:L1HS:REF
- ↕ chr2:143253223-143253225:+:L1HS:NONREF
- ↕ chr2:143860956-143866987:+:L1HS:REF
- ↕ chr2:148188745-148194773:+:L1HS:REF
- ↕ chr2:153007766-153013796:-:L1HS:REF
- ↕ chr2:157566353-157572377:+:L1HS:REF
- ↕ chr2:166988454-166994509:+:L1HS:REF
- ↕ chr2:169248623-169254656:+:L1HS:REF
- ↕ chr2:172315270-172321297:+:L1HS:REF
- ↕ chr2:175481951-175487994:-:L1HS:REF
- ↕ chr2:177973073-177979090:+:L1HS:REF
- ↕ chr2:180833661-180839689:-:L1HS:REF
- ↕ chr2:193170704-193171720:+:L1HS:REF
- ↕ chr2:193212420-193218448:-:L1HS:REF
- ↕ chr2:196905587-196911636:+:L1HS:REF
- ↕ chr2:198915189-198915191:+:L1HS:NONREF
- ↕ chr2:213567231-213573262:-:L1HS:REF
- ↕ chr2:230337069-230342513:-:L1HS:REF
- ↕ chr2:232149196-232155229:-:L1HS:REF
- ↕ chr3:3963076-3969110:+:L1HS:REF
- ↕ chr3:4916534-4922591:+:L1HS:REF
- ↕ chr3:11488672-11492490:-:L1HS:REF
- ↕ chr3:17858274-17861391:-:L1HS:REF
- ↕ chr3:18516080-18520244:+:L1HS:REF
- ↕ chr3:20707402-20707404:-:L1HS:NONREF
- ↕ chr3:26036528-26042769:-:L1HS:REF
- ↕ chr3:26398017-26404045:-:L1HS:REF
- ↕ chr3:29108111-29112113:-:L1HS:REF
- ↕ chr3:35454617-35458296:+:L1HS:REF
- ↕ chr3:46783105-46789138:-:L1HS:REF
- ↕ chr3:54394322-54400323:-:L1HS:REF
- ↕ chr3:55754551-55754553:+:L1HS:NONREF
- ↕ chr3:77763677-77769678:-:L1HS:REF
- ↕ chr3:80541011-80541013:-:L1HS:NONREF
- ↕ chr3:82095700-82095703:-:L1HS:NONREF
- ↕ chr3:82337499-82339442:+:L1HS:REF
- ↕ chr3:85527417-85527423:+:L1HS:NONREF
- ↕ chr3:89460825-89466856:-:L1HS:REF
- ↕ chr3:90169567-90175598:-:L1HS:REF
- ↕ chr3:103556537-103562569:-:L1HS:REF
- ↕ chr3:108749400-108755425:+:L1HS:REF
- ↕ chr3:109199872-109205903:+:L1HS:REF
- ↕ chr3:110888814-110890548:+:L1HS:REF
- ↕ chr3:116359999-116366026:+:L1HS:REF
- ↕ chr3:120573021-120579186:+:L1HS:REF
- ↕ chr3:123871878-123871880:+:L1HS:NONREF
- ↕ chr3:132946006-132952034:-:L1HS:REF
- ↕ chr3:135002247-135005976:-:L1HS:REF
- ↕ chr3:136479056-136485103:-:L1HS:REF
- ↕ chr3:136963693-136969736:-:L1HS:REF
- ↕ chr3:139590956-139596035:+:L1HS:REF
- ↕ chr3:151430754-151430756:-:L1HS:NONREF
- ↕ chr3:158019676-158025704:+:L1HS:REF
- ↕ chr3:159095379-159101394:-:L1HS:REF
- ↕ chr3:163236941-163242962:+:L1HS:REF
- ↕ chr3:164291525-164296426:+:L1HS:REF
- ↕ chr3:169552175-169552177:+:L1HS:NONREF
- ↕ chr3:186654351-186654353:+:L1HS:NONREF
- ↕ chr3:190964647-190965526:-:L1HS:REF
- ↕ chr4:14755114-14761144:+:L1HS:REF
- ↕ chr4:15841546-15847572:+:L1HS:REF
- ↕ chr4:16942776-16944630:+:L1HS:REF
- ↕ chr4:21159390-21165421:+:L1HS:REF
- ↕ chr4:23614771-23620793:+:L1HS:REF
- ↕ chr4:48051954-48057974:-:L1HS:REF
- ↕ chr4:52538471-52544498:+:L1HS:REF
- ↕ chr4:52816456-52822486:+:L1HS:REF
- ↕ chr4:57562316-57568347:-:L1HS:REF
- ↕ chr4:61939927-61945962:-:L1HS:REF
- ↕ chr4:62726948-62732969:-:L1HS:REF
- ↕ chr4:69664009-69667565:+:L1HS:REF
- ↕ chr4:70015396-70019676:-:L1HS:REF
- ↕ chr4:78105735-78111765:+:L1HS:REF
- ↕ chr4:79704552-79710581:+:L1HS:REF
- ↕ chr4:79937715-79943746:+:L1HS:REF
- ↕ chr4:87347103-87353146:-:L1HS:REF
- ↕ chr4:91437491-91443691:+:L1HS:REF
- ↕ chr4:91978211-91984413:+:L1HS:REF
- ↕ chr4:93608283-93614338:-:L1HS:REF
- ↕ chr4:93638307-93644337:-:L1HS:REF
- ↕ chr4:98592435-98598463:-:L1HS:REF
- ↕ chr4:106571057-106577070:-:L1HS:REF
- ↕ chr4:110690112-110693684:-:L1HS:REF
- ↕ chr4:111894801-111900831:+:L1HS:REF
- ↕ chr4:119948726-119954758:-:L1HS:REF
- ↕ chr4:135178140-135183747:-:L1HS:REF
- ↕ chr4:136293494-136299546:-:L1HS:REF
- ↕ chr4:137496278-137500000:+:L1HS:REF
- ↕ chr4:144809658-144810951:+:L1HS:REF
- ↕ chr4:146304143-146304145:+:L1HS:NONREF
- ↕ chr4:157986118-157986120:-:L1HS:NONREF
- ↕ chr4:166449405-166449678:-:L1HS:REF
- ↕ chr4:166569976-166576007:+:L1HS:REF
- ↕ chr4:166755895-166761908:+:L1HS:REF
- ↕ chr4:166850455-166851634:+:L1HS:REF
- ↕ chr4:169515501-169521532:-:L1HS:REF
- ↕ chr4:189137085-189143104:+:L1HS:REF
- ↕ chr5:4500001-4502372:-:L1HS:REF
- ↕ chr5:13416497-13422525:+:L1HS:REF
- ↕ chr5:15906515-15912550:-:L1HS:REF
- ↕ chr5:33797437-33797439:-:L1HS:NONREF
- ↕ chr5:34147845-34154031:+:L1HS:REF
- ↕ chr5:52086499-52088220:-:L1HS:REF
- ↕ chr5:58384174-58390206:+:L1HS:REF
- ↕ chr5:66750283-66754219:+:L1HS:REF
- ↕ chr5:79778884-79784938:-:L1HS:REF
- ↕ chr5:81616090-81622140:+:L1HS:REF
- ↕ chr5:86510690-86516743:+:L1HS:REF
- ↕ chr5:90154963-90154967:+:L1HS:NONREF
- ↕ chr5:102131356-102137385:+:L1HS:REF
- ↕ chr5:102189483-102194435:+:L1HS:REF
- ↕ chr5:104518587-104524616:+:L1HS:REF
- ↕ chr5:105513236-105519274:-:L1HS:REF
- ↕ chr5:109259387-109265418:+:L1HS:REF
- ↕ chr5:110144555-110144557:-:L1HS:NONREF
- ↕ chr5:111302238-111308262:+:L1HS:REF
- ↕ chr5:119684785-119690814:-:L1HS:REF
- ↕ chr5:133583288-133589299:+:L1HS:REF
- ↕ chr5:146609485-146615534:+:L1HS:REF
- ↕ chr5:152886441-152892473:-:L1HS:REF
- ↕ chr5:153070982-153077008:-:L1HS:REF
- ↕ chr5:156061919-156067966:-:L1HS:REF
- ↕ chr5:160709608-160715639:-:L1HS:REF
- ↕ chr5:162530742-162531677:-:L1HS:REF
- ↕ chr5:165068136-165068138:-:L1HS:NONREF
- ↕ chr5:166141191-166145692:-:L1HS:REF
- ↕ chr5:166966760-166972815:-:L1HS:REF
- ↕ chr5:173402796-173408828:-:L1HS:REF
- ↕ chr5:177772245-177778274:+:L1HS:REF
- ↕ chr6:2417774-2423803:-:L1HS:REF
- ↕ chr6:13502816-13502818:+:L1HS:NONREF
- ↕ chr6:19764892-19770918:+:L1HS:REF
- ↕ chr6:32621789-32621791:+:L1HS:NONREF
- ↕ chr6:51874783-51880802:+:L1HS:REF
- ↕ chr6:69495362-69500000:+:L1HS:REF
- ↕ chr6:70010347-70016552:+:L1HS:REF
- ↕ chr6:72988654-72994686:-:L1HS:REF
- ↕ chr6:76875759-76877075:-:L1HS:REF
- ↕ chr6:83333952-83339981:+:L1HS:REF
- ↕ chr6:86000000-86005073:-:L1HS:REF
- ↕ chr6:93865738-93871783:-:L1HS:REF
- ↕ chr6:94197995-94204060:-:L1HS:REF
- ↕ chr6:98703059-98708329:+:L1HS:REF
- ↕ chr6:102398209-102398211:-:L1HS:NONREF
- ↕ chr6:112703745-112709778:+:L1HS:REF
- ↕ chr6:117102131-117108163:+:L1HS:REF
- ↕ chr6:121162716-121168725:+:L1HS:REF
- ↕ chr6:128998385-129000000:+:L1HS:REF
- ↕ chr6:133020691-133026746:-:L1HS:REF
- ↕ chr6:142128962-142128964:-:L1HS:NONREF
- ↕ chr6:152712429-152712656:-:L1HS:REF
- ↕ chr6:156034135-156040165:+:L1HS:REF
- ↕ chr6:156324980-156331010:-:L1HS:REF
- ↕ chr6:157547703-157547705:-:L1HS:NONREF
- ↕ chr7:7465092-7471120:+:L1HS:REF
- ↕ chr7:8351723-8353975:+:L1HS:REF
- ↕ chr7:8847084-8847086:+:L1HS:NONREF
- ↕ chr7:12497211-12500000:+:L1HS:REF
- ↕ chr7:25041860-25047891:-:L1HS:REF
- ↕ chr7:30439242-30445274:+:L1HS:REF
- ↕ chr7:49680245-49686300:-:L1HS:REF
- ↕ chr7:53584807-53584809:-:L1HS:NONREF
- ↕ chr7:57539590-57540834:+:L1HS:REF
- ↕ chr7:63148831-63154859:-:L1HS:REF
- ↕ chr7:66286853-66292884:-:L1HS:REF
- ↕ chr7:70197328-70203357:+:L1HS:REF
- ↕ chr7:91173443-91179517:+:L1HS:REF
- ↕ chr7:93787624-93793679:-:L1HS:REF
- ↕ chr7:97613656-97619688:+:L1HS:REF
- ↕ chr7:108188818-108188820:+:L1HS:NONREF
- ↕ chr7:110707004-110713024:-:L1HS:REF
- ↕ chr7:111243515-111249546:-:L1HS:REF
- ↕ chr7:111963193-111969223:+:L1HS:REF
- ↕ chr7:113776122-113782152:-:L1HS:REF
- ↕ chr7:125179570-125181978:-:L1HS:REF
- ↕ chr7:141062014-141068042:-:L1HS:REF
- ↕ chr7:141920659-141926712:-:L1HS:REF
- ↕ chr7:147842591-147848623:-:L1HS:REF
- ↕ chr7:150597094-150602628:+:L1HS:REF
- ↕ chr8:8470859-8476906:+:L1HS:REF
- ↕ chr8:27113618-27119645:+:L1HS:REF
- ↕ chr8:75621331-75627355:-:L1HS:REF
- ↕ chr8:88685705-88691760:+:L1HS:REF
- ↕ chr8:91522091-91528121:-:L1HS:REF
- ↕ chr8:104739851-104745873:-:L1HS:REF
- ↕ chr8:121329878-121334807:-:L1HS:REF
- ↕ chr8:127311575-127315431:+:L1HS:REF
- ↕ chr8:134070756-134076773:-:L1HS:REF
- ↕ chr8:135875862-135881890:+:L1HS:REF
- ↕ chr8:136438074-136444105:-:L1HS:REF
- ↕ chr9:1447164-1447166:+:L1HS:NONREF
- ↕ chr9:5491417-5491419:+:L1HS:NONREF
- ↕ chr9:18560890-18560892:-:L1HS:NONREF
- ↕ chr9:20655632-20658802:-:L1HS:REF
- ↕ chr9:63913382-63916426:+:L1HS:REF
- ↕ chr9:75474002-75474004:-:L1HS:NONREF
- ↕ chr9:83049539-83055571:-:L1HS:REF
- ↕ chr9:90149604-90155634:+:L1HS:REF
- ↕ chr9:94113535-94119565:-:L1HS:REF
- ↕ chr9:95697585-95703604:+:L1HS:REF
- ↕ chr9:99498741-99500000:+:L1HS:REF
- ↕ chr9:103237485-103241471:+:L1HS:REF
- ↕ chr9:108802688-108802690:+:L1HS:NONREF
- ↕ chr9:110417102-110423126:+:L1HS:REF
- ↕ chr9:110791097-110797129:+:L1HS:REF
- ↕ chr9:112798107-112804159:-:L1HS:REF
- ↕ chr10:5245354-5251383:+:L1HS:REF
- ↕ chr10:19088601-19094618:-:L1HS:REF
- ↕ chr10:25418756-25418758:-:L1HS:NONREF
- ↕ chr10:33510845-33516876:-:L1HS:REF
- ↕ chr10:37995443-38000000:+:L1HS:REF
- ↕ chr10:67189566-67189568:+:L1HS:NONREF
- ↕ chr10:81441665-81441975:-:L1HS:REF
- ↕ chr10:85355506-85361538:+:L1HS:REF
- ↕ chr10:98782941-98788971:-:L1HS:REF
- ↕ chr10:105775520-105781551:-:L1HS:REF
- ↕ chr10:108310130-108316139:-:L1HS:REF
- ↕ chr10:122695714-122695716:+:L1HS:NONREF
- ↕ chr11:24327951-24334001:+:L1HS:REF
- ↕ chr11:25500009-25502139:-:L1HS:REF
- ↕ chr11:31315654-31321680:-:L1HS:REF
- ↕ chr11:36551606-36557636:-:L1HS:REF
- ↕ chr11:48847694-48853724:-:L1HS:REF
- ↕ chr11:54849946-54851079:+:L1HS:REF
- ↕ chr11:78677772-78683802:-:L1HS:REF
- ↕ chr11:82155865-82161891:+:L1HS:REF
- ↕ chr11:83941305-83941307:-:L1HS:NONREF
- ↕ chr11:85324758-85330821:+:L1HS:REF
- ↕ chr11:87340031-87346061:-:L1HS:REF
- ↕ chr11:90400067-90406098:-:L1HS:REF
- ↕ chr11:90966271-90972302:-:L1HS:REF
- ↕ chr11:93136638-93142673:+:L1HS:REF
- ↕ chr11:95436216-95442246:+:L1HS:REF
- ↕ chr11:108685166-108685556:-:L1HS:REF
- ↕ chr11:109177494-109183526:-:L1HS:REF
- ↕ chr11:125536609-125542640:+:L1HS:REF
- ↕ chr12:3500000-3505228:-:L1HS:REF
- ↕ chr12:33864388-33864390:-:L1HS:NONREF
- ↕ chr12:38799646-38805673:+:L1HS:REF
- ↕ chr12:44108220-44114234:-:L1HS:REF
- ↕ chr12:51562631-51568657:-:L1HS:REF
- ↕ chr12:54788573-54794627:+:L1HS:REF
- ↕ chr12:55096256-55102283:-:L1HS:REF
- ↕ chr12:69773410-69779441:-:L1HS:REF
- ↕ chr12:73283667-73289668:-:L1HS:REF
- ↕ chr12:74874868-74880901:+:L1HS:REF
- ↕ chr12:101146043-101152064:+:L1HS:REF
- ↕ chr12:108996947-109000000:+:L1HS:REF
- ↕ chr12:126299023-126305038:-:L1HS:REF
- ↕ chr13:19496786-19500000:+:L1HS:REF
- ↕ chr13:31302314-31308370:+:L1HS:REF
- ↕ chr13:34480262-34486294:-:L1HS:REF
- ↕ chr13:48465555-48471566:+:L1HS:REF
- ↕ chr13:54572234-54573121:+:L1HS:REF
- ↕ chr13:74426326-74432354:+:L1HS:REF
- ↕ chr13:76612823-76618851:-:L1HS:REF
- ↕ chr13:92685561-92691592:-:L1HS:REF
- ↕ chr13:97667408-97673440:-:L1HS:REF
- ↕ chr13:105382665-105383251:-:L1HS:REF
- ↕ chr13:108510472-108516495:-:L1HS:REF
- ↕ chr14:18283818-18289864:-:L1HS:REF
- ↕ chr14:30681601-30681603:-:L1HS:NONREF
- ↕ chr14:30684809-30690837:-:L1HS:REF
- ↕ chr14:44705080-44711104:-:L1HS:REF
- ↕ chr14:45477110-45483169:-:L1HS:REF
- ↕ chr14:46727484-46727758:-:L1HS:REF
- ↕ chr14:48187082-48193113:+:L1HS:REF
- ↕ chr14:51794601-51800632:-:L1HS:REF
- ↕ chr14:52201038-52201040:-:L1HS:NONREF
- ↕ chr14:58753700-58753702:+:L1HS:NONREF
- ↕ chr14:61055116-61061148:-:L1HS:REF
- ↕ chr14:62535851-62541882:+:L1HS:REF
- ↕ chr14:63116706-63122735:-:L1HS:REF
- ↕ chr14:70547290-70553322:+:L1HS:REF
- ↕ chr14:79314060-79314943:+:L1HS:REF
- ↕ chr15:47215130-47215132:-:L1HS:NONREF
- ↕ chr15:51417216-51423246:-:L1HS:REF
- ↕ chr15:54926081-54932099:-:L1HS:REF
- ↕ chr15:55958953-55958956:+:L1HS:NONREF
- ↕ chr15:81995166-82000000:+:L1HS:REF
- ↕ chr15:82882881-82888919:-:L1HS:REF
- ↕ chr15:83450804-83456834:-:L1HS:REF
- ↕ chr15:87509891-87515920:+:L1HS:REF
- ↕ chr16:9584490-9590522:-:L1HS:REF
- ↕ chr16:16840517-16846556:-:L1HS:REF
- ↕ chr16:32587481-32588397:-:L1HS:REF
- ↕ chr16:33952564-33958612:-:L1HS:REF
- ↕ chr16:34118474-34119193:+:L1HS:REF
- ↕ chr16:35608475-35614501:-:L1HS:REF
- ↕ chr16:54042096-54048145:+:L1HS:REF
- ↕ chr16:65690011-65696020:+:L1HS:REF
- ↕ chr16:68583448-68589505:-:L1HS:REF
- ↕ chr16:80727089-80730717:+:L1HS:REF
- ↕ chr16:83637252-83643296:+:L1HS:REF
- ↕ chr17:9615985-9622015:+:L1HS:REF
- ↕ chr17:66596579-66602595:-:L1HS:REF
- ↕ chr17:69000000-69005148:-:L1HS:REF
- ↕ chr17:70361267-70361269:+:L1HS:NONREF
- ↕ chr17:70458956-70464987:+:L1HS:REF
- ↕ chr17:70544788-70550795:-:L1HS:REF
- ↕ chr18:535701-541755:+:L1HS:REF
- ↕ chr18:4477329-4478177:+:L1HS:REF
- ↕ chr18:5684668-5687891:+:L1HS:REF
- ↕ chr18:13975860-13981891:-:L1HS:REF
- ↕ chr18:37819737-37825798:+:L1HS:REF
- ↕ chr18:41775014-41781045:+:L1HS:REF
- ↕ chr18:44021279-44024826:-:L1HS:REF
- ↕ chr18:47660373-47666427:+:L1HS:REF
- ↕ chr18:50343959-50349987:-:L1HS:REF
- ↕ chr18:61512674-61515526:-:L1HS:REF
- ↕ chr18:62906292-62912314:+:L1HS:REF
- ↕ chr18:67603633-67605600:+:L1HS:REF
- ↕ chr18:70746549-70752581:-:L1HS:REF
- ↕ chr18:72966526-72972556:-:L1HS:REF
- ↕ chr18:75846851-75852883:+:L1HS:REF
- ↕ chr19:43864494-43867300:-:L1HS:REF
- ↕ chr20:7116194-7122199:-:L1HS:REF
- ↕ chr20:11632779-11638837:+:L1HS:REF
- ↕ chr20:12801017-12807044:-:L1HS:REF
- ↕ chr20:39977475-39977785:+:L1HS:REF
- ↕ chr20:55859566-55865521:+:L1HS:REF
- ↕ chr22:15214621-15220667:-:L1HS:REF
- ↕ chr22:22131969-22131971:-:L1HS:NONREF
- ↕ chr22:22361253-22361257:+:L1HS:NONREF
- ↕ chr22:28059912-28059914:-:L1HS:NONREF
- ↕ chr22:28663283-28669315:+:L1HS:REF
- ↕ chr22:34549683-34555708:-:L1HS:REF
- ↕ chr22:48985761-48991792:-:L1HS:REF
- ↕ chrX:11707248-11713279:+:L1HS:REF
- ↕ chrX:23238516-23244575:+:L1HS:REF
- ↕ chrX:26314417-26320446:-:L1HS:REF
- ↕ chrX:50019456-50025505:-:L1HS:REF
- ↕ chrX:54118685-54124744:-:L1HS:REF
- ↕ chrX:56695884-56701916:+:L1HS:REF
- ↕ chrX:64013267-64019286:-:L1HS:REF
- ↕ chrX:73106142-73112170:+:L1HS:REF
- ↕ chrX:73380991-73387013:+:L1HS:REF
- ↕ chrX:76233354-76239154:+:L1HS:REF
- ↕ chrX:76322775-76328806:-:L1HS:REF
- ↕ chrX:80905077-80905430:-:L1HS:REF
- ↕ chrX:81841153-81847184:+:L1HS:REF
- ↕ chrX:83059584-83065637:-:L1HS:REF
- ↕ chrX:83542396-83548420:+:L1HS:REF
- ↕ chrX:83838051-83839860:-:L1HS:REF
- ↕ chrX:85733685-85734499:+:L1HS:REF
- ↕ chrX:87821407-87827419:+:L1HS:REF
- ↕ chrX:92254241-92256469:-:L1HS:REF
- ↕ chrX:96057824-96063842:-:L1HS:REF
- ↕ chrX:106469285-106475319:+:L1HS:REF
- ↕ chrX:112314520-112314522:-:L1HS:NONREF
- ↕ chrX:119435468-119441493:-:L1HS:REF
- ↕ chrX:127362223-127368248:+:L1HS:REF
- ↕ chrX:130517377-130523407:+:L1HS:REF
- ↕ chrX:142477849-142483853:+:L1HS:REF
- ↕ chrX:147653734-147659767:+:L1HS:REF
- ↕ chrX:149178166-149184185:-:L1HS:REF
- ↕ chrX:151330320-151336351:+:L1HS:REF
- ↕ chrX:155516016-155522048:-:L1HS:REF
- ↕ chrY:3443550-3449565:+:L1HS:REF
- ↕ chrY:4948913-4954938:-:L1HS:REF
- ↕ chrY:5606144-5612199:-:L1HS:REF
- ↕ chrY:9941130-9947151:+:L1HS:REF
- ↕ chrY:19034631-19038436:+:L1HS:REF

Methylation pattern visualisation created with
methpat version 2.1.0.
